# Fully automated fiber-based optical spectroscopy system for use in a clinical setting

**DOI:** 10.1117/1.JBO.23.7.075003

**Published:** 2018-07-06

**Authors:** Adam Eshein, Andrew J. Radosevich, Bradley Gould, Wenli Wu, Vani Konda, Leslie W. Yang, Ann Koons, Seth Feder, Vesta Valuckaite, Hemant K. Roy, Vadim Backman, The-Quyen Nguyen

**Affiliations:** a Northwestern University, Department of Biomedical Engineering, Evanston, Illinois, United States; b University of Chicago Medicine, Center for Endoscopic Research and Therapeutics, Chicago, Illinois, United States; c Boston Medical Center, Department of Gastroenterology, Boston, Massachusetts, United States

**Keywords:** fiber-optics, spectroscopy, medical optics, automated systems, tissue optics

## Abstract

While there are a plethora of *in vivo* fiber-optic spectroscopic techniques that have demonstrated the ability to detect a number of diseases in research trials with highly trained personnel familiar with the operation of experimental optical technologies, very few techniques show the same level of success in large multicenter trials. To meet the stringent requirements for a viable optical spectroscopy system to be used in a clinical setting, we developed components including an automated calibration tool, optical contact sensor for signal acquisition, and a methodology for real-time *in vivo* probe calibration correction. The end result is a state-of-the-art medical device that can be realistically used by a physician with spectroscopic fiber-optic probes. We show how the features of this system allow it to have excellent stability measuring two scattering phantoms in a clinical setting by clinical staff with ∼0.5% standard deviation over 25 unique measurements on different days. In addition, we show the systems’ ability to overcome many technical obstacles that spectroscopy applications often face such as speckle noise and user variability. While this system has been designed and optimized for our specific application, the system and design concepts are applicable to most *in vivo* fiber-optic-based spectroscopic techniques.

## 1 Introduction

In the last three decades, there have been hundreds of *in vivo* studies using optical spectroscopy for a myriad of applications.^1^^–^^9^ Many of the instruments in those studies have been fiber-optic-based probes, which can be extraordinarily robust, flexible, relatively cheap, and easy to assemble. While there have been many studies showing promising results in a number of different applications, currently there are few FDA-approved fiber-based optical spectroscopy techniques, which have been implemented in a clinical setting for diagnostic applications. This is in part, due to the many technical challenges of creating an optical spectroscopy instrument that is viable for adoption into mainstream clinical practice. In this work, we present tools that can overcome three of those technical challenges: robust and standardized calibration, automated *in vivo* signal acquisition, and real-time correction to *in vivo* signal acquisition.

Like any electro-optical instrument, fiber-optic probe systems have an optical transform function (OTF) that must be measured and accounted for to isolate the intrinsic signal of interest from tissue. The OTF can change from probe-to-probe, system-to-system, and with time. Typically, this is accounted for by calibrating the system with a number of standard samples with known optical properties. For a clinically viable system, the calibration process must be robust enough so it can be performed by physicians, nurses, and other medical staff, without extensive training or disruption to the normal clinic workflow. An automated calibration protocol is ideal.

Calibration measurements can be used to remove the OTF from a tissue optical measurement; however, there are also other concerns to consider. User measurement technique, including the pressure, angle, and time of contact, can all affect the measured optical signal.^1^^,^^10^^–^^12^ Ruderman et al.^10^ and Reif et al.^11^ showed that pressure from an optical probe can alter the biomarker under investigation and specifically alter the extracted parameters characterizing the organization of microvasculature. Here lies one of the biggest technical challenges for fiber-optic probes and the field of “optical biopsy” technologies. The promise of these techniques is their relative noninvasive and nonperturbing nature. However, several studies have shown that the particular use of fiber-optic probes directly interferes with the biomarkers under investigation. To standardize signal acquisition technique and ensure measured biomarkers are a reflection of the tissue under investigation, rather than a marker of some extrinsic factor, our group invented a tool to automate the signal acquisition from an optical probe without modification to the hardware.^13^

Another challenge that fiber-optic probes face during *in vivo* use is bending and twisting of the probe. These movements can put stress on the optical fibers and change their OTF. If this change occurs after the calibration measurements, then it can be impossible to properly remove the OTF from the measured signal by each fiber. To overcome this challenge, our group developed a method to measure each collection fibers’ relative throughput in real-time during tissue measurement acquisition. This allows correction for changes in the fibers’ OTF induced by bending during *in vivo* signal acquisition.

The optical spectroscopy system and its individual components presented in this work have been optimized for use with low-coherence enhanced backscattering spectroscopy (LEBS) but are widely applicable to other fiber-optic techniques. LEBS is an optical spectroscopy technique that our group invented and has developed for *in vivo* early detection of three separate cancers.^7^^,^^8^^,^^14^ LEBS is capable of measuring subdiffuse (source–detector separations less than a transport mean free path) and diffuse (source–detector separations greater than a transport mean free path) spectrally resolved backscattered light. LEBS has been described in detail in numerous other publications.^15^^–^^19^ To make this work accessible to a larger audience, optical parameters that are unique to LEBS are not calculated or shown. Instead all data and analysis shown in this work use the diffuse backscattering spectrum, which can be measured by a variety of probe types.^20^ LEBS is only used as a case in point to demonstrate the optical spectroscopic system. The principles that motivated the design of the system are universal to most *in vivo* optical spectroscopy techniques.

In this work, we present the features incorporated into the optical spectroscopy system allowing it to have improved robustness. We present the specific designs and methodology of our automated calibration tool, signal acquisition technique, and real-time OTF correction algorithm. We then present data showing the robustness in data that these features provide. The overarching goal of these tools is to automate the use of a spectroscopic fiber-optic device and to ensure the robustness of acquired data.

## 2 Materials and Methods

### 2.1 Automated Calibration

The goal of any optical spectroscopy device is to have a measurement that is completely sensitive to the intrinsic sample properties of interest. Thus, sensitivity to extrinsic properties such as the light source, optical fibers’ condition, spectrometer light throughput, and detector quantum efficiency must be removed. Therefore, every spectroscopy system requires several calibration steps. Typically, there are three extrinsic components of any sample measurement that need to be removed via calibration. (1) The internal reflections and background signal caused by the geometry of the device, (2) the spectral shape and intensity of the light source, and (3) the throughput and quantum efficiency of each collection channel and illumination channel. This is represented in Eq. (1), where $S_{\text{measured}}$ is the signal measured by the detector, L is the spectrum of the illumination source, B is the background response signal caused by internal reflections and electrical noise, $T_{\text{illumination}}$ is the throughput response of the illumination channel, $T_{\text{collection}}$ is the throughput response and quantum efficiency of the collection channel and detector, and $S_{\text{tissue}}$ is the intrinsic tissue response signal under investigation

$$S_{\text{measured}}=L(B+T_{\text{illumination}}\times T_{\text{collection}}\times S_{\text{tissue}}).$$ (1)

Thus, there are three traditional calibration steps needed: (1) background measurement to be subtracted from the sample measurement, (2) a standard measurement to normalize the sample signal to (remove effect of illumination source), and (3) flat-field to normalize each channel or sensor in the system. However, in practice, the number of calibration steps is actually much greater. For example, the bias or electrical background noise of the system may need to be measured and subtracted from the measurements. It is also recommended to have at least one optically scattering phantom measurement to monitor the state of the system, especially if multiple systems are being used in multiple clinics by multiple operators.^21^ This means there can be five or more calibration steps. Thus, the tissue measurement will actually be dependent on proper use of the system throughout the five calibration steps in addition to the tissue measurement. Given that the optical system is being operated in a setting that is not optimized for use of a delicate optical instrument (i.e., a typical doctor’s office or medical examination room), there is a high chance of a mistake in the calibration of the optical spectroscopy system and that mistake will manifest in the extracted sample signal. This could lead to a mistake in patient diagnosis and inappropriate treatment.

Therefore, we built and tested an automated calibration tool. This tool has three advantages: (1) repeatable and accurate calibration measurements that are independent of the operator, (2) no extensive training or expertise with optical systems required for use and no disruption to clinic work-flow, and (3) confirmation of robust calibration and tracking of system performance. This automated calibration tool has many unique components that are described in detail in the following sections. Specific dimensioned designs of the device are available from the corresponding author upon reasonable request. The device was designed for use with a 3.4-mm-diameter probe; however, it can easily be adapted to larger or smaller probes using different sized calibration standards and holders. It is important to ensure that the usable surface of each calibration standard is large enough to accommodate the functional area of the probe tip. For example, the presented flat-field fixture can maintain spatial homogeneity over a 1-mm-diameter surface as shown in Sec. 3.1.2.

#### 2.1.1 Automation and design

**Fig. 1:**
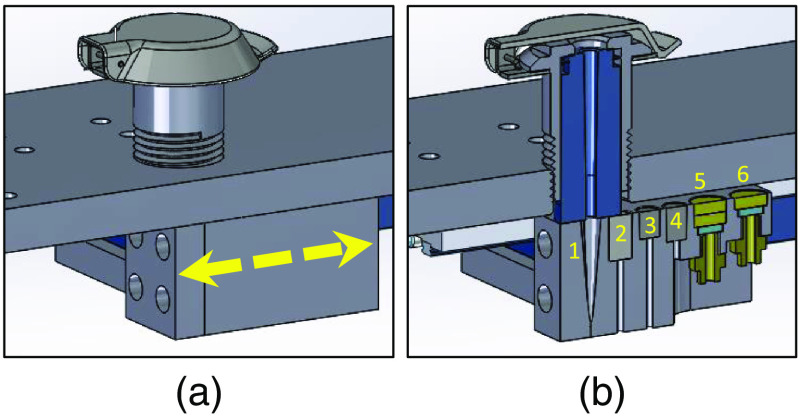
Automated calibration tool. (a) The upper component of the tool holds a fiber-optic probe, shown with a metallic cap closed. The lower component holding the calibration fixtures moves on a motorized stage under the probe holder. (b) Inside the lower component of the tool, six calibration fixtures are held. They are: (1) background, (2) white reflectance standard, (3, 4) optically scattering phantoms, (5) flat-field, and (6) Hg–Ar lamp.

A three-dimensional rendering of the automated calibration tool is shown in Fig. 1. The tool uses a motorized stage (MTS50-Z8, ThorLabs) connected to a customized fixture holding six unique calibration components. These components are a background fixture, a white reflectance standard, a flat-fielding standard, a wavelength calibration lamp fixture, and two optically scattering phantoms. Each of these components will be described in detail. To use the fixture, the fiber-optic probe is inserted into the top of the fixture and pushed down until the probe hits a stopper. The stopper holds the probe 0.2 mm above the fixtures. This prevents the probe from touching any of the substrates directly and damaging them. A computer controls the movement of the stage and acquisition of measurements from each calibration fixture. The stage automatically moves each calibration standard under the probe for measurements to be acquired.

#### 2.1.2 Background geometry

A rendering of the background calibration design is shown in Fig. 1(b). The fixture uses a specialized black absorbing material that absorbs 99.99% of incident light between 400 and 800 nm (Spectral Black Foil, Acktar, Israel). The fixture has a cone geometry. This design is similar to that tested by Breneman who showed that such a design can be used to maximize the number of reflections the light will have to undergo before returning to the source.^22^

In addition to subtracting the background signal from each measurement, it is also important to subtract the spectrometer bias or base level signal, as well as any external lighting that may enter the probe. We accomplish this by closing a shutter (Fiber Optic Switch, Avantes) in-line with the optical probe and light-source, and capturing a second measurement after each acquisition during all *in vivo* measurements, as well as all calibration measurements. This measurement captures the dark noise of the detector and any light originating external to the probe, without capturing any optical signal from the probe itself. Subtracting the bias signal is especially important when using nontemperature-controlled detectors, which is common for clinical applications due to their smaller design and lower cost. These nontemperature-controlled detectors have a read-in noise that can vary with temperature and time. For endoscopic applications, it is essential to subtract out the endoscope illumination light.

It should be noted that the *in vivo* and calibration measurement in these studies used relatively long accumulation times (250 to 1000 ms) and therefore were not affected by high-frequency oscillating external lighting. For applications using shorter acquisition times, the proposed method may not be suitable for removing the effects of external lighting.

#### 2.1.3 Transmission flat-field design

A rendering of the flat-field fixture design is shown in Fig. 1(b). The design utilizes two diffusers (Opal Diffusing Glass, Edmund Optics) that are facing opposite directions, to maximize the spacing between the diffusing surfaces. Light from a 1-mm-diameter multimode optical fiber (M35L01, ThorLabs) is passed through the diffusers on to the tip of the probe. This geometry optimizes spatial homogeneity of the light. Thus, each channel in the fiber-optic probe will receive an equal amount of radiance.

As discussed in Sec. 2.1.2, it can be beneficial to subtract the detector bias signal from all measurements. This is especially important for the flat-field calibration measurement. Using the proposed flat-field design, the probe light source is turned off and light enters the probe from an external source, i.e., the flat-field fixture. Therefore, there is no background signal to subtract; however, the flat-field signal still contains a bias signal that does not give meaningful information about the throughput of the optical system. Thus, this bias signal should be subtracted to properly use the flat-field calibration measurement.

It should be noted that the fibers’ throughput (or OTF) can change with bending and movement. This can become a problem in applications when fiber-optic probes must be bent between calibration and *in vivo* signal acquisition. To correct for any changes induced in the fibers’ OTF after the flat-field calibration, we developed the real-time flat-field correction algorithm described in Sec. 2.2.

#### 2.1.4 Wavelength calibration feature

**Fig. 2:**
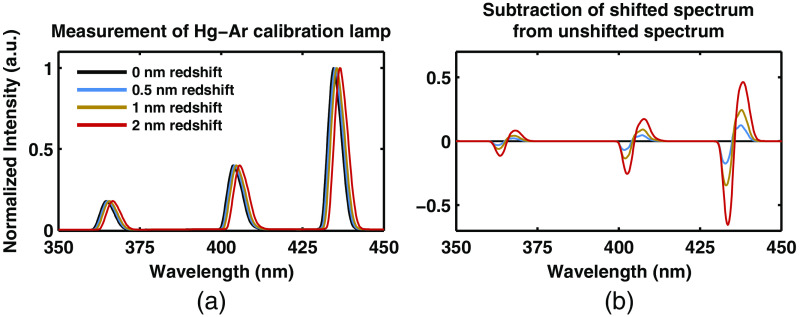
Measurement of Hg–Ar calibration lamp. (a) The measurement of the Hg–Ar calibration lamp with increasing wavelength calibration shifts. (b) The direct subtraction of the nonshifted spectra with the shifted spectra.

Many optical spectroscopy techniques require processing (e.g., subtraction) of spectra, measured from different channels or detectors,^5^^,^^18^^,^^23^^,^^24^ or fitting wavelength-dependent features in the spectrum.^23^^,^^25^^–^^28^ If the wavelength calibration on the detectors is not accurate or has shifted with time since initial factory calibration, then small features in the spectra can become large artifacts in the resulting processed spectra. This process is shown in Fig. 2. A spectrum of a mercury–argon (Hg–Ar) lamp (HG-1, Ocean Optics) is shown in Fig. 2(a) along with spectra measured with flawed wavelength calibration so that they are redshifted. Figure 2(b) shows the result of subtracting the uncalibrated redshifted spectra from the true spectra. This process creates large artifacts in the spectra. Therefore, it is important to track the wavelength calibration of all detectors with each calibration by measuring a calibration lamp. Moreover, it is important to track the wavelength calibration with each calibration to monitor the changing state of the spectrometers in real-time and catch hardware failures before they have a negative impact on data. Due to its characterized spectrum in the ultraviolet, visible, and near-infrared range, we chose a Hg–Ar lamp (HG-1, Ocean Optics). The design of this fixture is shown in Fig. 1(b). It consists of a 200-μm fiber delivering light from the Hg–Ar lamp through a single diffuser (Opal Diffusing Glass, Edmund Optics).

#### 2.1.5 Reflectance standard and scattering phantoms

In the automated calibration tool, three optically scattering phantoms are measured. A 99% reflective Spectralon white standard (WS-1, Ocean Optics) is measured and used to normalize all sample measurements. A commercial solid phantom (Biomimic Optical Phantom, INO, Canada) and a custom-made silicone phantom created using a method similar to Bays et al.,^29^ with known scattering parameters, are measured to track the robustness of each calibration sequence. Use of such phantoms is essential for tracking the stability and performance of spectroscopic instruments used in clinical research trials.^21^^,^^30^ Measurement of the phantoms allows confirmation of robust calibration and gives confidence in tissue measurements that may otherwise have appeared aberrant. It also gives a reliable metric to use to remove data points from a study, rather than just removing all outlier measurements. Furthermore, with real-time analysis of the phantom measurement, calibration errors can be caught before the tissue measurement even begins. The user can be prompted to repeat the calibration or even check for a hardware failure (e.g., a damaged optical fiber).

Since these are solid samples, there is a random speckle pattern in the spectral measurement of these standards. This will be particularly true in any spectroscopy application utilizing a coherent or partially coherent light source (e.g., a laser).^31^^,^^32^ Therefore, the automated calibration tool allows measurements from several locations on the solid phantoms to take an ensemble average of many unique random speckle patterns. This is accomplished by moving the linear stage a user-defined distance after each single accumulation. Multiple accumulations are then averaged together to remove the effect from speckle.

### 2.2 Real-Time Flat-Field

As previously discussed, it is necessary with many probe designs to acquire a “flat-field” calibration to compensate for different optical channels’ throughput. The fixture presented in Sec. 2.1.3 is an ideal design to accomplish this; however, in certain applications (e.g., endoscopic use), a probe may undergo bending and twisting that can alter the OTF of the fibers by >1.5%. In this case, the flat-field calibration acquired before the tissue measurements does not take into account changes in the fibers OTF induced by the movement of the probe between the calibration measurements and tissue measurement. Therefore, we developed a method for real-time flat-field correction to overcome this problem.

**Fig. 3:**
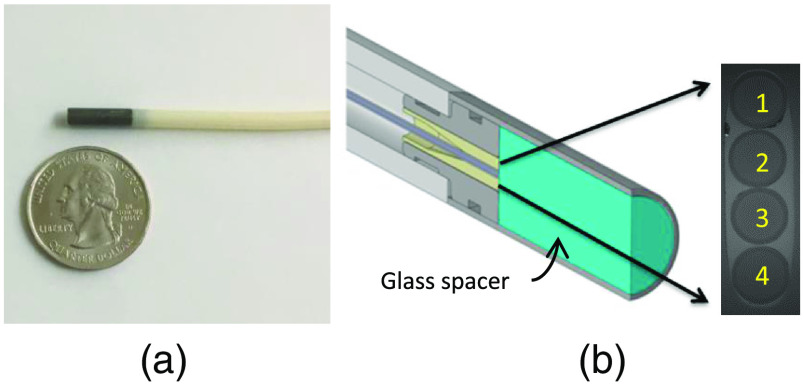
Fiber-optic probe used for studies. (a) The fiber-optic probe. (b) A schematic of the design of the probe tip with a blow up en-face view of the fibers. The fibers have a core diameter of 50  μm and a center-to-center fiber spacing of ∼60  μm. A 9-mm scratch resistant glass spacer with a beveled surface (9.5 deg) separates the fibers from the medium under investigation.^18^

The technique we have developed gives the relative difference in signal between collection channels with no calibration, other than background signal subtraction. Figure 3 shows an optical probe with a symmetrical fiber geometry. Figure 3(b) shows a schematic of the probe with four fibers in the probe. The real-time flat-field correction method works by illuminating the outer fibers sequentially, and then collecting the two inner fibers simultaneously during each of the outer fiber illumination periods. This allows measuring of the same signal by two different fibers, which then allows removal of the OTF of each individual fiber. Note that the assumption made in this method is that each fiber is sampling the same area of tissue (identical optical properties). The probe used in these studies has a 9-mm glass spacer that allows the fibers to have at least 93% overlapping sampling geometries. For probes with nonoverlapping geometries, the expected spatial variances in optical properties of the medium under investigation must be considered.

When fiber 1 is illuminated, then fiber 2 measures a signal that can be expressed as

$$S21_{\text{measured}}=(L)(T1_{\text{illumination}})(T2_{\text{collection}})(S\alpha_{\text{tissue}}),$$ (2)

and fiber 3 measures a signal that can be expressed as

$$S31_{\text{measured}}=(L)(T1_{\text{illumination}})(T3_{\text{collection}})(S\beta_{\text{tissue}}),$$ (3)

where L is the spectrum of the illumination source, $S21_{\text{measured}}$ and $S31_{\text{measured}}$ are the signals measured on the spectrometer from fibers 2 and 3, respectively, $T2_{\text{collection}}$ and $T3_{\text{collection}}$ are the transfer functions for fibers 2 and 3, respectively, $T1_{\text{illumination}}$ is the transfer function of the illumination channel 1, and $S\alpha_{\text{tissue}}$ and $S\beta_{\text{tissue}}$ are the intrinsic tissue response signals measured by fibers 2 and 3, respectively, that are to be isolated.

When fiber 4 is illuminated, Eqs. (2) and (3) become Eqs. (4) and (5)

$$S24_{\text{measured}}=(L)(T4_{\text{illumination}})(T2_{\text{collection}})(S\beta_{\text{tissue}}),$$ (4)

and

$$S34_{\text{measured}}=(L)(T4_{\text{illumination}})(T3_{\text{collection}})(S\alpha_{\text{tissue}}).$$ (5)

The key differences in these equations are that fiber 4 is now the illumination fiber, and most importantly, fibers 2 and 3 have switched which intrinsic tissue response signals they are measuring.

We can isolate the intrinsic sample signals $S\alpha_{\text{tissue}}$ and $S\beta_{\text{tissue}}$ by combining these four measurements into

$$\frac{S\alpha_{\text{tissue}}}{S\beta_{\text{tissue}}}=\sqrt{\frac{\left(S21_{\text{measured}})(S34_{\text{measured}}\right)}{\left(S24_{\text{measured}})(S31_{\text{measured}}\right)}}.$$ (6)

Note that this equation is only valid after background subtraction is applied to each measurement, i.e., $S21_{\text{measured}}$ must have a background calibration measurement with the fiber 1 as illumination and fiber 2 as collection subtracted from it. Such a background measurement must be acquired and subtracted for all four measured signals. These background measurements are acquired during the automated calibration sequence described in Sec. 2.1.2. Otherwise, no other calibrations are needed to isolate $S\alpha_{\text{tissue}}/S\beta_{\text{tissue}}$. This equation can be generalized for any symmetric fiber design. To isolate the relative difference between two intrinsic sample signals, two measurement channels that have two illumination channels that are equal distances from the two respective collection channels are needed, i.e., the distance from channels 1 and 2 must be equal to the distance between 3 and 4.

### 2.3 Automated In Vivo Measurement Acquisition

It has been shown that *in vivo* optical reflectance measurements using a fiber-optic probe can be highly dependent on the measurement technique of the user. This results from the optical signal being sensitive to the probe’s contact pressure with tissue, angle of contact, and length of time in contact with the tissue.^10^^–^^12^ To remove the influence of these factors, we developed an automated acquisition algorithm. This algorithm can be applied to most fiber-based *in vivo* optical spectroscopy techniques without modification to the hardware. The algorithm is described in detail by Ruderman et al.^13^ In brief, the algorithm operates by sampling the optical reflectance signal continuously with relatively short accumulation time (e.g., 10 ms) at a user-defined wavelength. The collected signal is normalized to a white reflectance standard signal (acquired during the calibration process) in real time. Once the normalized signal rises above a predefined threshold and remains stable (<3% variability) for a user-defined number of consecutive accumulations, the system activates for a full spectral measurement (e.g., >300  ms). After the measurement is complete, the algorithm waits for the reflected intensity to drop below a second user-defined threshold, indicating that the probe has been removed from the tissue surface. At this point, the algorithm will “reset” waiting for the reflected intensity to rise above the first user-defined threshold signaling solid contact with tissue for the next measurement.

### 2.4 Collection of In Vivo Clinical Data

The studies presented in this work were approved by the Institutional Review Board at the University of Chicago Medical Center (Chicago, Illinois). Patients were eligible for recruitment into the study if they were already scheduled for population-based colonoscopy screening or surveillance as recommended by their general practitioner or gastroenterologist. A total of 14 asymptomatic patients that were free of colorectal cancers were recruited into the studies after providing written informed consent.

**Fig. 4:**
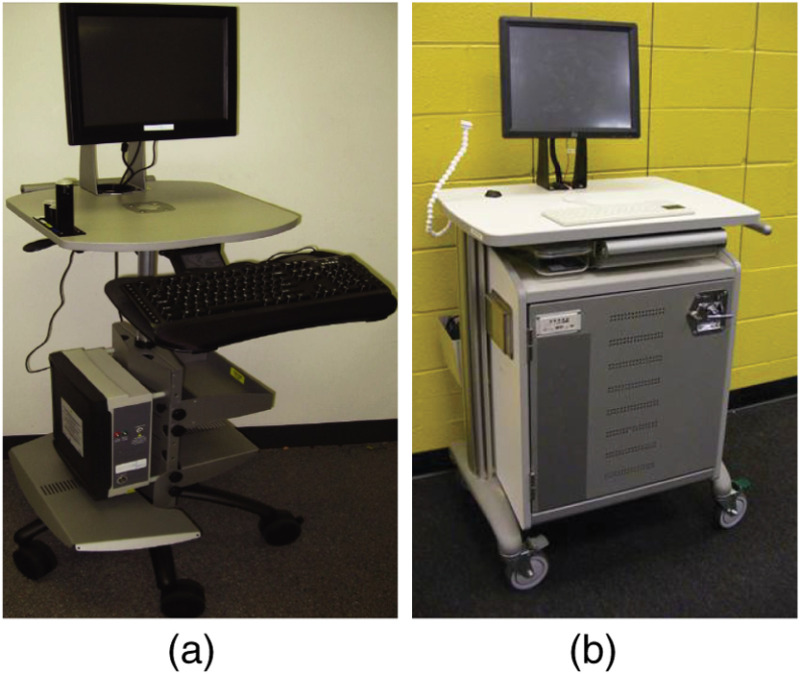
Point-of-care optical spectroscopy instruments. (a) Portable cart housing a computer system with the fiber-based spectrometers (USB 2000, Ocean Optics), broadband light source (HPX 2000, Ocean Optics) and manually operated calibration mechanism. (b) Portable optical spectroscopy instrument housing three spectrometers (Maya LSL, Ocean Optics), broadband light source (HPX 2000, Ocean Optics), medical grade battery power source, and computer system, which fully automates the entire system.

All measurements were acquired through a point-of-care instrument shown in Fig. 4(a) (assembled by Tricor Systems) or a fully automated point-of-care instrument shown in Fig. 4(b) (assembled by Garrett Technologies). A 3.4-mm-diameter LEBS probe was introduced into the rectal vault via a custom introducer that allows blind insertion of the probe without causing discomfort to the patient. The measurements were taken immediately before a colonoscopy and digital rectal exam on patients who had undergone standard bowel preparation. The probe operator then took at least 10 measurements from random locations within the rectum, applying gentle contact with the tissue surface. Each of the 10 measurements was acquired with a 500-ms accumulation time and included a subsequent measurement with an in-line shutter between the light source and probe closed that was subtracted from the initial measurement. This allowed for subtraction of the spectrometer bias as well as any external room light collected by the probe, as described in Sec. 2.1.2. The entire procedure from probe insertion to extraction typically took <2  min. Measurements were acquired by trained endoscopists and medical research specialists, and the final data analysis was performed by the investigators using automated data analysis algorithms.

## 3 Results

### 3.1 Automated Calibration Unit

#### 3.1.1 Background fixture

**Fig. 5:**
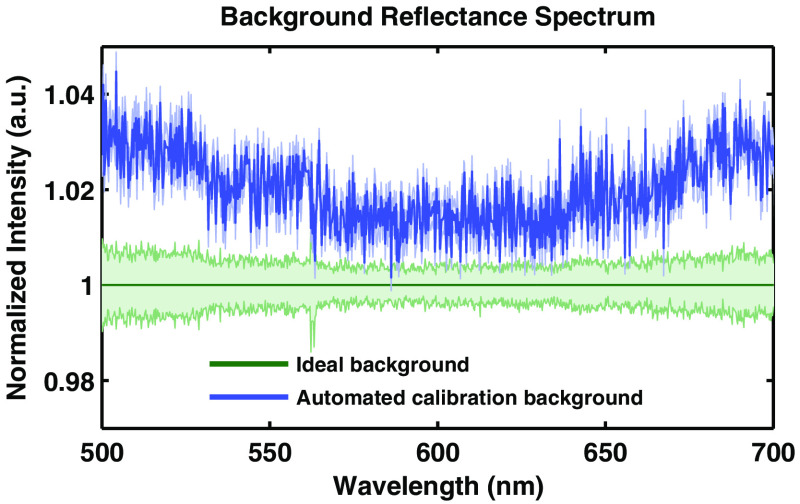
Background calibration. Comparison of an ideal background measurement, with that measured by the automated calibration tool.

The goal of the background component of the automated calibration fixture is to mimic an ideal background measurement. An ideal background measurement only measures internal reflections inside the probe and electrical noise of the detector and does not measure any returned light from outside the probe. This can be experimentally achieved by acquiring a measurement in a dark room with no external lighting, with the probe directed at a very distant, nonreflective surface. Unfortunately, it is not practical to acquire such a measurement in a clinic every time the probe is to be used. Thus, we created a background calibration fixture that mimics the effect of an ideal background measurement. Figure 5 shows that the measurement from the background fixture is on average <3% higher than the ideal background measurement, indicating that the fixture allows measurement of the internal reflections of the probe and not reflected light from the fixture itself. This demonstrates that the calibration measurement is an excellent proxy for an ideal background measurement, and the fixture is very small with a volume $<0.5\;{\mathrm{cm}}^{3}$.

#### 3.1.2 Transmission flat-field fixture

**Fig. 6:**
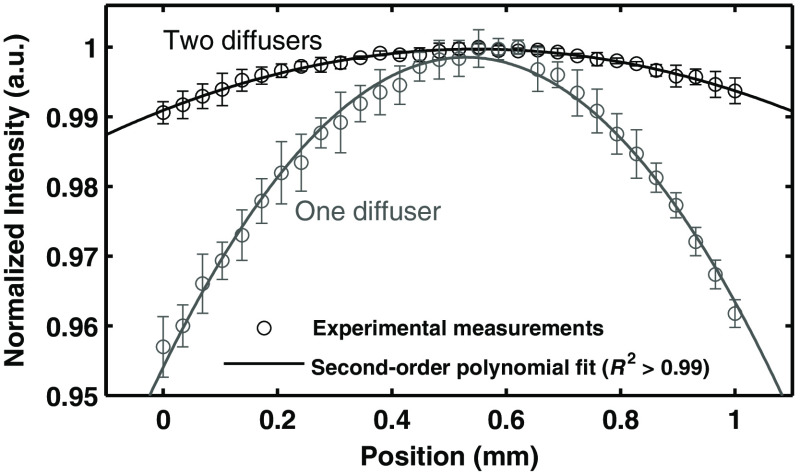
Flat-field calibration. The mean intensity as a function of position over the center of the flat-field fixture, measured ∼9.2  mm away from the fixture. A fixture design with one and two diffusers are compared.

We developed a flat-field calibration fixture that utilizes two off-the-shelf diffusers with their diffusing surfaces facing outward to optimize the total diffusing capability of the fixture. The ideal flat-field fixture should deliver an equal amount of light to all channels of an optical device. To accomplish this, the fixture must have a uniform spatial distribution of light. We experimentally characterized this by moving an optical probe across the surface of the fixture. We measured intensity as a function of position across the surface of the fixture, at fixed distance of ∼9.2  mm away from the fixture surface. This allows evaluation of the spatial homogeneity of the fixture. Figure 6 shows there is <1% fluctuation in the intensity of light across the center 1 mm of the fixture, indicating a nearly uniform spatial distribution of light. We also tested a fixture design with a single diffuser and show that it has >4% fluctuation across the center 1 mm of the fixture. This demonstrates the benefit of the two diffuser design.

#### 3.1.3 White reflectance standard fixture

**Fig. 7:**
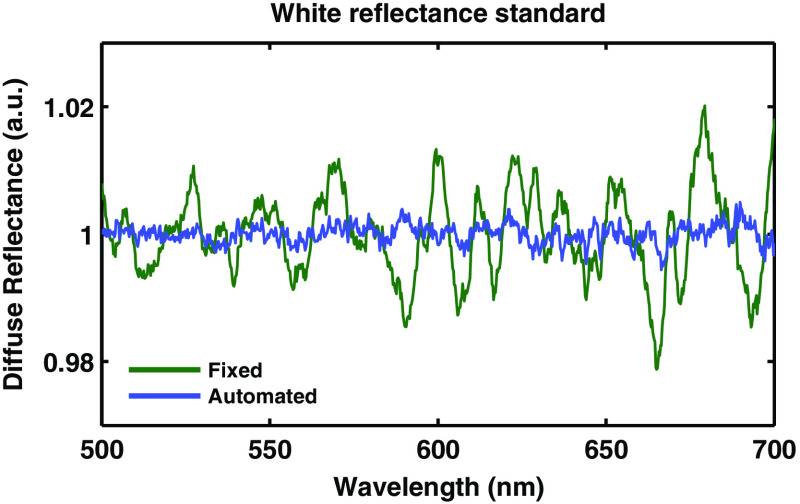
White reflectance standard calibration. A comparison of acquiring diffuse reflectance measurements from a fixed measurement and an automated, moving measurement. Both spectra shown are the average of 20 single accumulations. For both sets of measurements, the illumination spot was ∼1  mm in diameter.

Many spectroscopic techniques that utilize a fully or partially coherent light source suffer from speckle noise.^31^^,^^32^ In this study, a broadband xenon lamp (HPX 2000, Ocean Optics) was used as the illumination source. According to Van Cittert–Zernike theorem, the illumination light gains partial spatial coherence as it travels through the 9-mm glass rod.^18^ The temporal coherence is determined by the spectral resolution of the spectrometer (0.25 nm).^33^ Figure 7 shows the diffuse reflectance spectrum of a white reflectance standard (WS-1, Ocean Optics). The spectra in green in Fig. 7 shows the effect of speckle noise on the diffuse reflectance spectrum. However, this noise can be overcome by measuring reflectance measurements from the sample at several, unique locations. This can be accomplished with the automated calibration tool by simply moving the linear stage small increments after each individual measurement. The spectra in blue in Fig. 7 show the effect of taking an ensemble-average of 20 measurements with unique speckle patterns, at unique locations on the surface of the reflectance standard. While both spectra have 20 averaged accumulations, there is more noise in the traditional, fixed measurement, which has ∼20 times higher spectral variance than the automated measurement. Both measurements are normalized by an ideal measurement that is an average of 300 measurements acquired while moving the probe over the surface of a large white reflectance standard phantom. In this case, we can consider all speckle noise to be eliminated by the ensemble-averaging.

#### 3.1.4 Scattering phantom fixtures

**Fig. 8:**
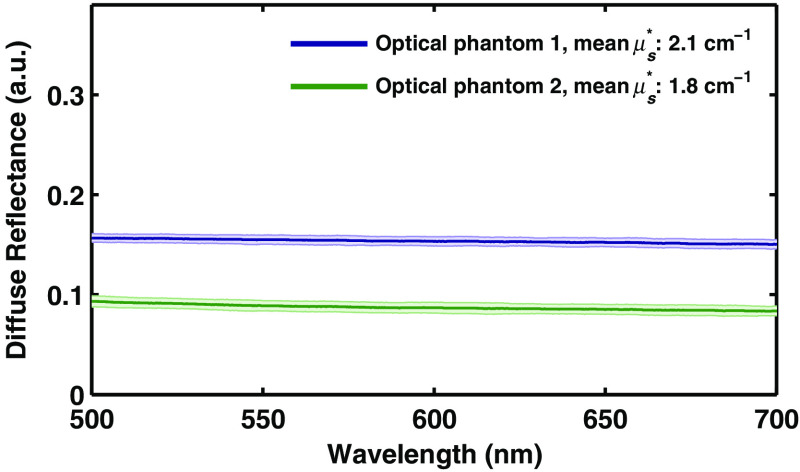
Diffuse reflectance spectrum from optically scattering phantoms. Measurements from 25 unique calibrations performed in a clinical setting, before the device was used for *in vivo* measurements in a patient. Error bars show standard deviation.

Figure 8 shows the reflectance spectrum from 25 unique calibrations performed in a clinical setting, on different days, with two different fiber-optic probes, and by three clinical users (nontechnical users). As can be seen in the figure, the system is extraordinarily robust with the standard deviation being 0.004 and 0.005 for each phantom (0.4% and 0.5% of the white standard phantom). Following a single 10-min training session, technicians performed subsequent measurements unsupervised in a clinical setting immediately prior to *in vivo* tissue measurements.

### 3.2 Automated In Vivo Signal Acquisition

Automating the process of *in vivo* signal acquisition is essential to ensure ease-of-use for the user and to guarantee the robustness of the optical measurement. Making secure contact with tissue, using consistent pressure, and consistent timing between touching tissue and triggering a measurement can be difficult for the user and take special expertise (e.g., surgical instrument or endoscopy training). It has been shown that the technique of the user can greatly influence the tissue properties extracted from the tissue.^10^^–^^12^

**Fig. 9:**
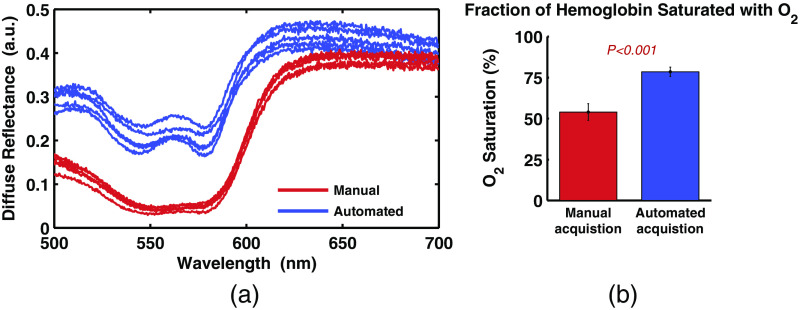
Demonstration of *in vivo* automated acquisition. (a) Comparison of five measurements acquired manually and five measurements acquired using the automated acquisition algorithm from one patient. (b) Comparison of the extracted fraction of oxygen-saturated hemoglobin from manual and automated measurements on 14 patients. Error bars show standard error.

Figure 9(a) shows a comparison of five measurements acquired using automated signal acquisition algorithm, and five measurements acquired manually from a single patient, on the rectal mucosa tissue. For the manual measurements, a trained endoscopist operated the probe and informed a technician when to trigger the measurement acquisition (pressing a button on the optical spectroscopy system). One of the advantages of the automated measurement algorithm is that it forces the user to completely retract the probe from the tissue surface after each measurement. This should increase the likelihood of sampling unique tissue locations with each measurement. In Fig. 9(a), the five manual measurements appear very similar, suggesting that they may have been repeatedly acquired from the same tissue location. On the other hand, the automated measurements show more variability, which may be the result of sampling more unique tissue locations within the rectum. Note that the trend of manual measurements having lower intrapatient variability is nonsignificant (P>0.05) when looking at all patient data.

Figure 9(b) shows comparison of manual and automated measurements from 14 patients. The fraction of hemoglobin saturated with oxygen was extracted from the diffuse backscattering spectrum using methods described in other publications.^26^^,^^27^ Measurements acquired manually have a lower percentage of oxygen-saturated hemoglobin, and despite the manual measurements in Fig. 9(a) appearing very similar, the interpatient variance is significantly higher (P<0.05) indicating a level of inconsistency from patient-to-patient. The results presented here suggest that manual measurements could be sensitive to the user’s (endoscopist) technique and are less consistent. The automated acquisition measurements have significantly (P<0.05) lower interpatient variance and a higher average oxygen saturation that is within the expected physiological range and agrees with previous investigations of oxygen saturation measured in human rectal mucosa.^34^ All measurements were performed on prepped patients, before a colonoscopy procedure, as described in Sec. 2.4.

### 3.3 Real-Time Flat-Field Correction

During *in vivo* use, fiber-optic probes can often undergo severe bending and twisting. These movements can alter the transmission efficiency of the optical fibers by >1.5%. To overcome this, we developed a method of recovering the relative throughput of optical channels in symmetric fiber-optic probes, as described in Sec. 2.2. This method relies on two sequential measurements of all collection channels, with two different illumination channels. After background subtraction, Eq. (6) is applied to the signals for these two sets of measurements to extract the ratio between the intrinsic tissue response signal measured by each fiber. This ratio can be converted to an absolute measurement, by normalizing to a calibration standard as described in Sec. 2.1.5.

**Fig. 10:**
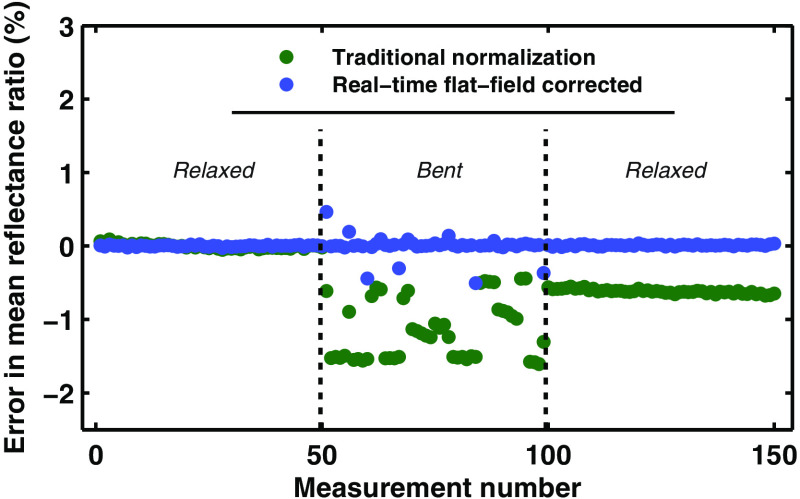
Demonstration of the real-time flat-field correction algorithm. The green points show a direct division of the collection channel with the shorter SDS by the channel with the larger SDS. The blue points show the data processed using Eq. (6) to correct for changes in the fibers’ OTF induced by bending.

Figure 10 shows the results of using the probe shown in Fig. 3 with and without the real-time flat-field correction method. The experiment was conducted by securing the probe tip at a fixed distance from a white standard phantom (WS-1, Ocean Optics) and continuously acquiring reflectance measurements from two collection channels. During the continuous measurements, the probe body was bent and deformed as it might be during an *in vivo* endoscopic procedure. Figure 10 shows comparison of the data when utilizing the real-time flat-field correction algorithm described in Eq. (6), and when doing the more basic processing of simply normalizing the fiber with the shorter source–detector separation (SDS) by the fiber with the longer SDS. As can be seen in the figure, the real-time flat-field correction algorithm maintains the correct ratio between the two collection channels with ∼18 times lower error. The only instances of error are during the brief moment while the probe is being moved. This occurs because the fibers’ OTF is actually different between the two sequential measurements. Once the probe stops moving, two sequential measurements can be acquired that accurately correct for the fibers’ newly modified OTF. However, the traditional processing of the data shows significant errors induced by bending. Of note, even when the probe is unbent and allowed to return to its relaxed position, there is still a persistent error in the measured ratio between the two collection channels.

## 4 Discussion and Conclusions

**Fig. 11:**
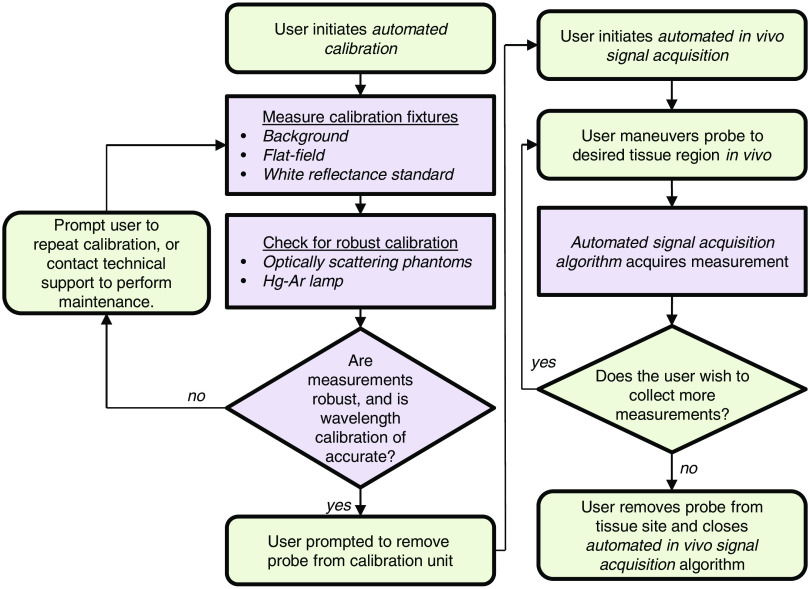
Flowchart describing operation of the automated optical spectroscopy system. Steps in green require user interaction, and steps in purple are fully automated.

In this work, we show the design, methodology, and results from unique tools used in a fiber-optic, optical spectroscopy system for *in vivo* tissue characterization. These tools aim to increase ease of use while also increasing the stability and accuracy of the *in vivo* optical measurement. These aims are accomplished through the automation of the entire use of the spectroscopy system from calibration to *in vivo* measurement acquisition. A clinical optical spectroscopy system equipped with these features can be adopted more easily and effectively into mainstream clinical use. This has been a major challenge for the biomedical optics research community, despite many successful research clinical studies.^1^^–^^5^^,^^7^^,^^8^ Figure 11 shows a flowchart describing the use of the system from calibration, through *in vivo* measurement acquisition. Steps in purple are fully automated and require no interaction from the user.

The first of the tools we have presented is the automated calibration device. Calibration is essential when comparing measurements from different patients from different days in order to remove all extrinsic influences on the measured spectra. This device makes the calibration process easier and quicker for the user, while also improving the accuracy and stability of the system. Figures 5 and 6 show that the geometry and design of the background and flat-field fixtures are optimized to provide as close to an ideal measurement as possible. Figure 7 shows how averaging measurements acquired from unique locations on a solid phantom can overcome the speckle noise that often plagues optical technologies using coherent or partially coherent light sources.^31^^,^^32^ The automated calibration device accomplishes this by automatically moving the fixture a predefined distance after each initial measurement. To ensure proper calibration and ensure measurement robustness, two optically scattering phantoms and a Hg–Ar calibration lamp are measured. Figure 2 shows the importance of tracking the wavelength calibration of the spectrometers onboard the system. Small errors in the wavelength calibration can result in large artifacts in the extracted tissue signal. Finally, Fig. 8 shows the measured diffuse reflectance spectra from two solid phantoms, measured by different users on different days using the automated calibration device. The standard deviation of the measurements of both phantoms across different days with different probes and different users in a clinical setting is ≤0.5%, demonstrating the robustness of the automated calibration tool (note that phantom measurements are not intended to be representative of *in vivo* tissue measurements). Each calibration can be tracked for accuracy, and if a calibration attempt does not meet a given standard, the user can be prompted to repeat the calibration, or contact a technical expert to perform maintenance on the system. The Hg–Ar calibration lamp can be used to correct any errors of the spectral calibration of each spectrometer onboard the system. These sorts of functionalities are essential for optical spectroscopy systems that are used by many nontechnical personnel across different clinical locations.

After the calibration process is complete, the fiber-optic probe is ready for *in vivo* tissue measurements. For this process, we have invented a simple optical tissue contact sensor that requires no additional hardware. This contact sensor serves two purposes: (1) allow easy use of the probe for blind insertion and (2) ensure measurement robustness by removing the effect of a specific user’s measurement technique. Since the probe will automatically collect measurements when the probe reaches complete and stable contact with tissue, use of the probe does not require special training (e.g., endoscopy training). The system automatically alerts the probe user and begins measurement acquisition when the probe is in contact with tissue. To ensure stable contact and prevent measurement acquisition during sliding, the algorithm performs a stability check. Several short consecutive measurements are examined for stability before the full measurement acquisition begins. In addition, this tool allows for more consistent measurement of *in vivo* biomarkers as shown in Fig. 9. In summary, this tool allows for easy use of a fiber-optic probe while also ensuring measurement robustness by automating signal acquisition and checking for stability.

In cases where the probe is used for measurements that require significant bending and twisting of the probe, such as endoscopic use, we have developed an algorithm that corrects for errors induced in the measurement due to changes in the optical fibers’ OTF after the automated calibration is complete. Figure 10 shows the ability of the algorithm to recover the ratio of reflectance measurements between two fibers, during bending and motion. Without using this real-time flat-field correction method, information could never be recovered once the fibers have been bent.

As stated previously, all the tools and parameters presented in this work have been optimized for our group’s specific technology and application. However, the technical challenges that these tools seek to overcome are common to many *in vivo* spectroscopic techniques. Our approach to overcoming these challenges is to create a system that is fully optimized from the point of calibration, through the end of *in vivo* tissue measurements. The automated calibration tool allows for easy calibration and has optical phantom measurements that can be used for evaluating the robustness of the calibration process. The *in vivo* signal acquisition has also been automated to ensure ease of use while also ensuring robust measurements. The real-time flat-field correction algorithm corrects small errors induced in the measurement by bending. In the end, we try to minimize all ambiguity and variability of the optical system and measurement process.
